# Transactions of the Chicago Society of Physicians and Surgeons, August 10, 1874

**Published:** 1874-09-01

**Authors:** Ralph E. Starkweather


### Society Reports.

### TRANSACTIONS OF THE CHICAGO SOCIETY OF PHYSICIANS AND SURGEONS.

### REGULAR MEETING, AUGUST 10, 1874.

*Reported by Ralph E. Starkweather, M.D.*

DR. A. Fisher was, upon motion, called to preside, the two Presidents being unavoidably absent, when the hour for business arrived.

The names of Drs. H. N. Hurlbut and F. L. Wadsworth were proposed for membership, and referred to the Board of Censors. Dr. Etheridge read an exceedingly interesting article, which he had translated from the *Progres Medical*, being a paper by Dr. H. Chauppe on the Therapeutical Study of Ipecac when used in the Sweats of Pulmonary Phthisis. The paper will appear in full in a subsequent number of the EXAMINER.

Dr. D. A. K. Steele, of the County Hospital, reported the following four cases of land scurvy, treated in the medical wards of Cook County Hospital:

Case 1st.—E. M. J., aged thirty-five; cabinet-maker; native of Norway; admitted July 25th; states that he has been in this country nine years; suffered from rheumatism before coming to this country; was perfectly well on the voyage; for five years he enjoyed pretty good health, with the exception of an occasional attack of intermittent fever; for the past three or four years he has been boarding himself, living in the basement of a barn, it being a cold, damp and dirty room, and sleeping in his clothing on a bench; working at his trade when he could obtain anything to do; subsisting on bread and meat, with occasionally a glass of ale and porter. For the past two years his diet consisted almost exclusively of salt pork, ham, rye bread and coffee. He has eaten no vegetables whatever, and the low state of his finances precluded the use of the ale and porter. He was subject to melancholy and habitually low spirited, and paid no attention whatever to personal cleanliness. Six weeks ago he commenced to have pain in his back and side, and his arms and legs became stiff. The following week he began to have severe headache and constant thirst; his bowels became costive. Four weeks ago he noticed that his mouth became sore and his teeth loose, four of them falling out; he was unable to masticate his food; the gums became swollen and painful; had an offensive taste in his mouth.

On admission: The patient is of medium height, sanguineo-nervous temperament, and presents a pale, exsanguinated appearance; skin dry and harsh; pulse 88 per minute, weak and compressible; respiration 24 per minute; temperature 98°; appetite good; bowels costive, having moved only twice[illegible] the last four weeks; he urinat[illegible]eadily, the water being high [illegible]red and somewhat scanty; co[illegible]ains of pain and stiffness of [illegible]e legs, difficulty in mastication, [illegible] some pain in the chest and s[illegible]ness of breath. On examina[illegible]n, find the tongue pale and flabby; gums swollen and spongy, almost covering the teeth in some places, and bleeding very readily; breath very offensive. The lower extremities are covered with large blotches of ecchymosis, most marked posteriorly; there is also some degree of ecchymosis around the left eye. Below the knees the legs are covered with a petechial eruption; the limbs are somewhat œdematous, the serous infiltration of the tissues around the knee-joint interfering with perfect flexion and extension of the legs. On physical examination of the chest, find the heart somewhat atrophied; a soft blowing murmur follows the first sound, at both base and apex. The lung sounds are nearly normal. Expiration is slightly prolonged.

Case 2nd.—T. C., aged 8, schoolboy, U. S., admitted July 27th, states that he has been living in a Catholic Orphan Asylum for the past year. He was well until five weeks ago, when his gums became swollen, bleeding easily; teeth became loose, and the legs swollen and painful. His diet at school was composed of meat, bread, soup, &c., but vegetables were entirely excluded; slept in a clean, airy room, and bathed the entire body once daily.

On admission: The patient is a bright, well-nourished lad; skin rather sallow; tongue pale; appetite good; bowels costive, moving but once a week; stools dry and hard. On examination, find patient's gums swollen, spongy and soft, nearly covering the teeth; teeth loose; breath fetid; find the legs œdematous; blotches of ecchymosis about the popliteal spaces, and a purpuric eruption below the knees. For the past few weeks he has been troubled with incontinence of urine.

Case 3rd.—J. N., aged 9, schoolboy, U. S., admitted July 30th.

He has had the same diet and hygienic surroundings as the previous patient, being an inmate of the same institution. He was well until two weeks ago, at which time his mouth became sore; the gums became swollen and bled easily; the teeth became loose, two of them dropping out. Soon his right leg became stiff and he had pain in the knee, it becoming swollen, and ecchymosis occurring upon the posterior aspect of the leg. He felt weak, and easily became exhausted.

On admission: The patient is emaciated and anæmic in appearance; tongue pale; conjunctivæ pearly and bloodless; appetite fair; bowels costive, moving only twice in two weeks; pulse 120 per minute; respirations 28; temperature 98½°.

On examination, find the gums swollen, spongy and tender; right knee stiff and painful from serous and synovial infiltration about the joint; both legs are slightly œdematous, and covered with petechial spots below the knees.

Case 4th.—S. M., aged 8, schoolboy, U. S., admitted July 30th. He also had the same diet and hygienic surroundings as the the two previous patients. He was well until ten days ago, and since that time he has manifested symptoms similar to those of the previous patient.

On admission: Patient seems fairly nourished, but the tongue is pale and flabby and the conjunctivæ pearly. Complains of sore mouth; bleeding of the gums; looseness of the teeth; pain and stiffness about the knee-joints; appetite good; bowels regular. On examination, find legs and thighs œdematous and ecchymosed; knee-joints stiff; purpuric eruption below the knees.

These patients were ordered a vegetable diet, with acid drinks, and tincture of the chloride of iron. Locally, used a mouth wash of permanganate of potash, one grain to the ounce of water. Under this treatment they have steadily improved, the ecchymosis, œdema, purpuric eruption and stiffness of the legs gradually disappearing. They have attained a ruddy, healthy appearance, very much in contrast with the sallow, exsanguinated appearance they presented on admission. They have been allowed to go out and take plenty of exercise in the open air every day.

Considering, then, that scurvy is one of the most difficult diseases to cure, and noting the prompt alleviation of the symptoms in these cases, under the use of abundant vegetable diet, moderate exercise, and remedies calculated to supply the deficiencies existing in the blood, we present the cases above detailed as an addition to the literature of the subject.

Dr. Hyde brought before the Society the subject of prescribing proprietary medicines or remedies—a practice so largely on the increase in this city, that six out of nine of the prescriptions received by the average grade of drug stores (perhaps not so large a proportion by the best class of stores), ordered proprietary medicines, and even named the makers of the goods. He entered his protest against such practice, and against the swarms of traveling salesmen who throng into the offices, peddling their samples of medicines, repeating their glib stories with tiresome sameness. Thus they go the rounds, month after month, and leave their bottles behind them, hoping, by donating a few samples, to increase the demand for their goods. What is the effect upon the wholesale drug-dealer? His stock will be made up of *proprietary medicines*, one-sixth; *patent medicines*, one-third; *paints* and *oils*, one-third; and the balance, *one-sixth*, of *drugs* and *medicines for prescriptions*.

Dr. Hay suggested a remedy which he had persistently applied; he orders the drug peddler out of his office and throws the medicines out after him.

Mr. A. E. Ebert, a leading druggist of this city, Editor of the *Pharmacist*, and lately President of the American Pharmaceutical Association, was present, and, by request, addressed the Society substantially as follows:

Twenty years ago the wholesale druggists sold, in equal quantities, drugs and patent medicines. Fifteen years ago, the fluid extracts and preparations of the so-called "Elegant Pharmacy" became the fashion. In 1860, the U. S. Pharmacopia fixed the strength of fluid extracts, but no honest, conscientious pharmacist can prepare them and compete successfully with the dishonest manufacturers.

Mr. Ebert exposed the vagaries of the elixir and the sugar-coated pills business, and various adulterations. One of these consisted in taking the silver coin of the country and making the same into nitrate of silver: in one case he found one ounce of pure copper in thirteen of nitrate of silver. The makers of many articles of "Elegant Pharmacy" cannot, by any exertion, sell their goods in their own cities. The people are learning to do without physicians, and can go to the stores, look at the medicines and buy whatever they choose. It injures the physician and degrades the pharmacist—makes middlemen of them—and kills off all incentive to accurate, scientific, honorable pharmacy. The only way in which to quell this evil is to cease mentioning names of makers, and to order only the officinal remedies authorized by the U. S. Pharmacopia.

Dr. Hay said that the remarks of Dr. Hyde and Mr. Ebert suggested the relations which ought to exist between the druggist, pharmacist and physician. After referring to the attacks lately made by secular papers upon the profession, alleging that physicians were defrauding the public and their patients by collusion with druggists, from whom they received a commission on prescriptions and other perquisites, he said: "If these charges are true, it is quite time we should relieve ourselves of the odium; if false, let us refute them. I am interested in the profession—its standing, its integrity, honor, and the confidence it receives from the public, which is the sole basis on which it rests." He had made some investigations which had induced him to think that the charges, as made, were true. He would, therefore, introduce the following preamble and resolutions, which were passed without one negative vote, in a meeting of above the average attendance of members:

WHEREAS, The medical profession of Chicago, on several recent occasions, has been charged in the Chicago *Times* with collusion with pharmacists, for the purpose of extorting money from their patients; and,

WHEREAS, This collusion is alleged to be effected in various ways; such as

1. The use of prescription papers bearing the business cards of pharmacists.

2. The occupation of offices free of, or at nominal, rents adjacent to or belonging to pharmacists.

3. The writing of private formulæ understood by certain pharmacists exclusively.

4. The acceptance of commissions upon prescriptions from pharmacists; and,

WHEREAS, The practices above designated, although deprecated by many, always have been maintained by many others, innocently and in good faith, unsuspicious of their abusive application; therefore, be it

*Resolved*, That the Society of Physicians and Surgeons of the City of Chicago recognize the fact that the practices above designated tend to the degradation and demoralization of the medical profession, to the diminution and withdrawal of public confidence, upon which its existence depends.

*Resolved*, That the members of this Society pledge themselves, as individuals and as an organization, to discontinue the practices above designated, so far as they may have adopted them, and to discountenance them in others so far as their influence may extend.

*Resolved*, That the Society regards the acceptance of commissions upon prescriptions, by physicians from pharmacists, positively disreputable and dishonest, and to be deemed a sufficient cause for the rejection of an applicant for, or the expulsion of a holder of, membership herein.

Dr. Jackson, in seconding the adoption of the resolution, said that it was a growing evil, and so far as it rests with us, we should shake it off.

Dr. Hamill—I heartily concur with the sentiment of the resolution.

Dr. Etheridge—The evil is imaginary rather than a real one. He had called on a few of the leading druggists, such as Bliss & Sharp, Dyche, Sargent, Buck & Rayner, and found that they never allowed commissions. He was astonished to find so little of it. The *Times* reporter circulated among the lower classes of physicians and druggists: it is the charlatans who bring these charges upon us. Are we not fighting a phantom?

Following remarks by Drs. Hamill and A. Fisher, Dr. Jackson said he hoped the practice was much less than was alleged, but thought that where there was so much smoke there must be some fire, and alluded to an incident where a medical friend of his had a bottle of perfumery and a roll of bank bills presented to him by an apothecary as a commission for prescriptions. The money was indignantly returned, and his friend forever afterwards withheld his patronage from that druggist.

The following resolution was proposed by Dr. Hyde:

*Resolved*, That a committee of three be appointed by the chair to co-operate with a similar committee from the Chicago College of Pharmacy (should such be appointed), in order to consider what, if any, measures are requisite in order to correct the great and growing evil in our midst of the prescribing of proprietary medicines, by name or otherwise, and of the accepting of samples of such medicines as are distributed in this city by the agents of eastern wholesale drug-houses. And that such joint-committees (should concurrence be had) report in full to the Society for such action in the premises as may seem to it desirable.

Dr. Hay seconded the resolution.

Mr. Ebert was of the opinion that the College of Pharmacy would co-operate.

Dr. Wilder gave details of a case where a druggist, who had probably never seen the inside of a medical or pharmaceutical school, took charge of a labor case, but finding it a complicated one, became frightened, and sent purposely to a physician other than the one regularly attending the family.

The resolution was passed by an unanimous vote, and Drs. Hyde, Hay and Etheridge were appointed as such committee.

Dr. Jackson, on behalf of the Fee Bill Committee, reported a schedule of rates for fees. The Committee were directed to confer with a similar committee of the Chicago Medical Society.

Upon motion, the Society adjourned.

---

SANITARY NOTES.—The whole science of hygiene may be included in the one word CLEANLINESS. The removal of refuse of all kinds, solid, liquid, and gaseous, is embraced within it, and pure air and water become a necessary result of the operation. It is a trite saying, "Nature abhors a vacuum," or, more correctly, it may be said, Nature always *supplies* a vacuum. Whenever we remove foul matter, stagnant water, and superfluous dust, we admit air, and generally far purer air, and water, to take their places.—*Sanitarian for September.*

